# Supplementary material for: Effectiveness of Combinational Treatments for Alzheimer’s Disease with Human Neural Stem Cells and Microglial Cells Over-Expressing Functional Genes
Source: Int J Mol Sci. 2023 May 31;24(11):9561. doi: 10.3390/ijms24119561 (PMC10253978; doi:10.3390/ijms24119561)
Supplement: Supplementary file 1 [file ijms-24-09561-s001.zip › ijms-2360021-supplementary.pdf]

International Journal of *Molecular Sciences*

Manuscript ijms-2360021

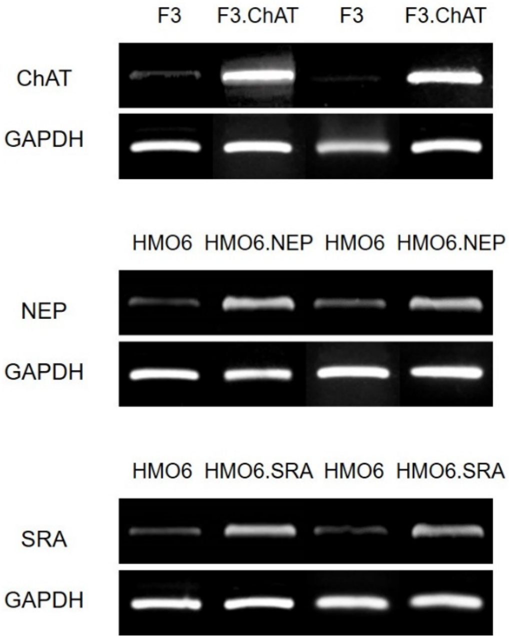

Supplementary Figure S1. RT-PCR analysis of mRNA of ChAT, NEP and SRA in the established cells, F3.ChAT, HMO6.NEP and HMO6.SRA, respectively. Primer sequences: ChAT forward: 5′-CTGTGCCCCCTTCTAGAGC-3′; reverse: 5′-CAAGGTTGGTGTCCCTGG-3’, NEP: forward: 5′-ATCAGCCTCTCGGTCCTTGT-3′; reverse: 5′-TGGAAGACAGCGCAAGACTC-3’, SRA forward: 5′-AGGAGATCGAGGTCCCACTG-3′; reverse: 5′-TGTTTCCACTCCCCTTTTCC-3’ for SRA.

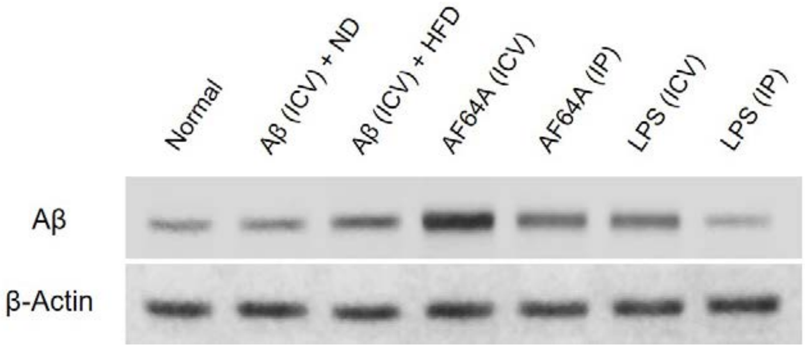

Supplementary Figure S2. Western blot analysis of brain Aβ using a primary antibody specific for Aβ and a secondary goat anti-rabbit IgG conjugated with horseradish peroxidase.

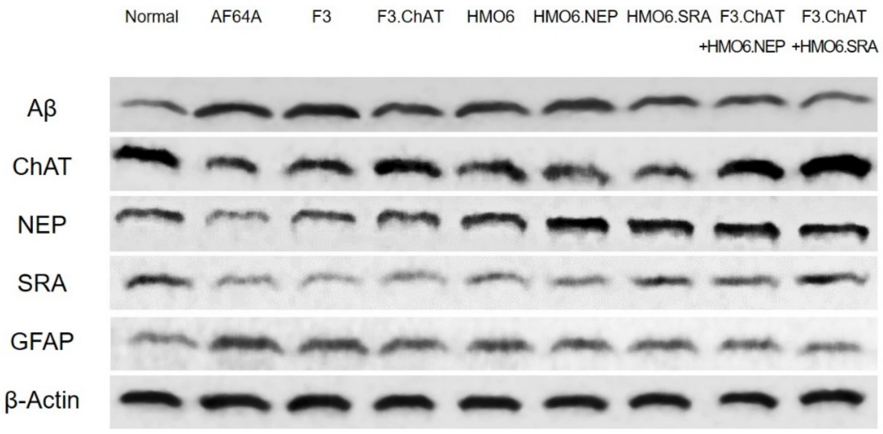

Supplementary Figure S3. Western blot analysis of brain Aβ functional proteins using primary antibodies specific for Aβ, ChAT, NEP, SRA or GFAP and secondary goat anti-rabbit IgG conjugated with horseradish peroxidase.

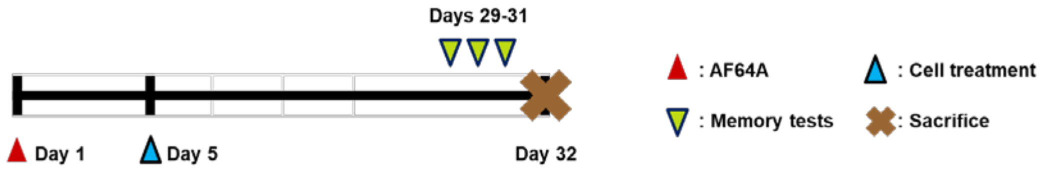

- Learning & memory tests: passive avoidance & water-maze performances
- ACh and Aβ concentrations: ELISA
- Survival and expression of functional genes of injected cells
  : immunohistochemistry [human mitochondria (hMito), ChAT, NEP, MSRA]
- Aβ & GFAP levels: western blotting

Supplementary Figure S4. Experimental design displaying the induction of cognitive dysfunction with AF64A, cell treatment, memory tests and analysis.
